# Ponicidin Inhibits Monocytic Leukemia Cell Growth by Induction of Apoptosis

**DOI:** 10.3390/ijms9112265

**Published:** 2008-11-19

**Authors:** Jia-Jun Liu, Yong Zhang, Wei-Bin Guang, Hong-Zhi Yang, Dong-Jun Lin, Ruo-Zhi Xiao

**Affiliations:** 1 Department of Hematology, The Third Affiliated Hospital of Sun Yat-sen University, Guangzhou, Guangdong 510630, P.R. China. E-Mails: jiajunliu2002@yahoo.com.cn (J. L.); lindongjun0168@163.com (D. L.); ruozhi_xiao@yahoo.com (R. X.); 2 Department of Nuclear Medicine, The Third Affiliated Hospital of Sun Yat-sen University, Guangzhou, Guangdong 510630, P.R. China. E-Mail: zy5040@163.com; 3 Department of Traditional Chinese Medicine, The Third Affiliated Hospital of Sun Yat-sen University, Guangzhou, Guangdong 510630, P.R. China. E-Mail: gwbing@pulic.guangzhou.gd.cn

**Keywords:** Ponicidin, Survivin, Bcl-2, Bax, Leukemia

## Abstract

In this study two monocytic leukemia cell lines, U937 and THP-1 cells, were used to investigate the anti-proliferation effects caused by ponicidin. Cell viability was measured by an MTT assay. Cell apoptosis was assessed by flow cytometry as well as DNA fragmentation analysis. Cell morphology was observed using an inverted microscope and Hoechst 33258 staining. RT-PCR and Western blot analysis were used to detect survivin as well as Bax and Bcl-2 expressions after the cells were treated with different concentrations of ponicidin. The results revealed that ponicidin could inhibit the growth of U937 and THP-1 cells significantly by induction of apoptosis. The suppression was in both time- and dose-dependent manner. Marked morphological changes of cell apoptosis were observed clearly after the cells were treated with ponicidin for 48∼72 h. RT-PCR and Western blot analysis demonstrated that both survivin and Bcl-2 expressions were down-regulated remarkably while Bax expression remained constant before and after apoptosis occurred. We therefore conclude that ponicidin has significant anti-proliferation effects by inducing apoptosis on leukemia cells *in vitro*, downregulation of survivin as well as Bcl-2 expressions may be the important apoptosis inducing mechanisms. The results suggest that ponicidin may serve as potential therapeutic agent for leukemia.

## 1. Introduction

Ponicidin, a diterpenoid compound, is extracted and purified from the traditional Chinese herbs Rabdosia rubescens or Isodon japonicas [1, 2]. Ponicidin has been reported to have a variety of biological effects such as immunoregulatory and anti-inflammatory functions as well as anti-viral functions especially in the upper respiratory tract infection [3]. Recent laboratory data suggest that ponicidin is a very effective anti-tumor agent, with profound effects on a number of cancer cells such as human leukemia cell line K562, human breast cancer cell line Bcap37, human gastric cancer cell line BGC823, human urinary bladder cancer cell lineBIU87, and Hela cell lines [1–4]. Later studies have shown that ponicidin can promote the cell-killing activity of antiherpes prodrugs acyclovir (ACV) and ganciclovir (GCV) in human cancer cells expressing herpes simplex virus thymidine kinase (HSV-TK), and the combined use of ponicidin with GCV or ACV may produce high cytotoxicity in viral TK-expressing cancer cells and lead to a rapid and enhanced tumor elimination *in vivo* [5]. New data have demonstrated that ponicidin can inhibit the growth and metastasis of prostate cancer due to its significant antiangiogenic activity [6].

Although ponicidin has been proved to be very effective in a variety of malignancies, many of its anti-tumor mechanisms remain to be demonstrated. Up to date, no detailed data are available about the role and mechanisms of ponicidin in leukemia cells. In order to understand the roles of ponicidin in leukemia cells and possible clinical application of ponicidin in leukemia therapy, we have investigated the effects of different concentrations of ponicidin on cell viability and apoptosis on monocytic leukemia cells *in vitro*.

## 2. Experimental

### 2.1. Main reagents

Ponicidin was kindly provided by Prof. XL Pan. Hoechst 33258 was purchased from Sigma Company. The antibodies used in this study, anti-Survivin, anti-Bcl-2 and anti-Bax, were purchased from Santa Cruz Company (Germany). TRIZOL was from GIBCO (USA), and reverse transcriptional kit was from MBI (USA); PCR primers (shown in Table 1) were synthesized by Shanghai Shenggong Company (China).

### 2.2. Cell culture

U937 and THP-1 cells were provided by the Central Laboratory of the Sun Yat-sen University Cancer Center. Normal peripheral blood monocytes (NPBMs) were isolated from healthy volunteers after obtaining informed consent by means of Ficoll density gradient centrifugation (specific gravity, 1.077; Shanghai Reagent Factory, Shanghai, China).Cells were cultured in RPMI-1640 medium supplemented with 10% fetal calf serum (FBS), 100U/mL penicillin and l00 μg/mL streptomycin, in a humidified incubator with 5% CO_2_ at 37 °C. All the cells were passaged twice weekly and routinely examined for mycoplasma contamination. Cells in logarithmic growth phase were used for further experiments.

### 2.3. Cell viability assay

The viability of the cells was assessed by MTT assay, which is based on the reduction of MTT by the mitochondrial dehydrogenase of intact cells to a purple formazan product. Briefly, U937 and THP-1 cells as well as NPBMs in logarithmic growth-phase were collected, and 2 × 10^5^ cells/well were dispensed within 96-well culture plates in 100 mL volumes. Then different concentrations of ponicidin (10, 20, 30, 40 and 50 μmol/L) were put in different wells. Every one of the concentrations above was regarded as one treated group while there was no ponicidin in the control group. Each of the treated or control group contained six parallel wells. Culture plates were then incubated for 0, 24, 48 and 72 hours prior to the addition of tetrazolium reagent. MTT working solution was prepared as follows: 5 mg MTT/mL PBS was sterile by being filtered with 0.45 μm filter units. Each of the above cultured wells was added 20 μL of MTT working solution and then incubated continuously for 4 hours. The water insoluble formazan was formed during incubation and it was solublized by adding solublization agent to each well. Amount of formazan was determined by measuring the absorbance at 540 nm using an ELISA plate reader.

### 2.4. Flow cytometry (FCM) detection

For FCM analysis, U937 and THP-1 cells treated with different concentrations of ponicidin for different times were collected, pelleted, washed with PBS, and resuspended in PBS containing 20 mg/L PI and 1g/L ribonuclease A. 1 × 10^6^ fixed cells were examined per experimental condition by flow cytometry. The percentage of degraded DNA was determined by the number of cells displaying subdiploid ( sub-G_1_) DNA divided by the total number of cells examined.

### 2.5. Detection of mRNA expressions using RT-PCR

The expressions of Survivin as well as Bcl-2 and Bax in U937 and THP-1 cells were examined by RT-PCR before and after the cells were treated with different concentrations of ponicidin for 72 h. The total RNA was extracted by using TRIZOL reagent according to the instructions described on the kit, which was certified to be suitable for RT-PCR by using agrose gel electrophoresis. First-stranded cDNA was synthesized using 5 μg total RNA by RT-PCR kit. The related PCR primers listed in Table 1 were used to produce the correlated products respectively.

As an internal control, the expression of β-actin was detected. The PCR reaction for β-actin cDNAs was performed with 30 amplification cycles and the reaction conditions were: denaturation at 94 °C for 1 min, annealing at 53 °C for 2 min, and extension at 72 °C for 3 min. The PCR reactions for both survivin and Bcl-2/Bax cDNAs were performed with 40 amplification cycles respectively as follows: (1) survivin and Bcl-2: denaturation at 94 °C for 45 s, annealing at 61 °C for 1 min, and extension at 72 °C for 1 min). (2) Bax: denaturation at 94 °C for 45 s, annealing at 58 °C for 1 min, and extension at 72 °C for 1 min. All of the above PCR reactions were incubated in an automatic heat-block(Model PJ 2000 DNA Thermal Cycler. The PCR products were then run on 1.5% agarose gel in TAE buffer for 20-30 min respectively and then visualized by ethidium bomide staining.

### 2.6. Hoechst 33258 staining

The morphology of U937 and THP-1 cells exposed to ponicidin for different time was observed firstly under inverted microscope. Then Hoechst 33258 staining was used to observe the apoptotic morphology. Cells were fixed with 4% formaldehyde in phosphate buffered saline (PBS) for 10 min, stained by Hoechst 33258 (10 mg/L) for one hour, and then subjected to fluorescence microscopy. After treatment with ponicidin, the morphologic changes including reduction in the volume and nuclear chromatin condensation were observed.

### 2.7. DNA fragmentation assay

Apoptosis was confirmed by detection of fragmentation of chromosomal DNA with the classic DNA ladder method. Briefly, 2 × 10^6^ cells were immersed in cytolysis buffer (Tris-HCL 1 mmol/L pH 8.0, edetic acid 10 mmol/L pH 8.0, proteinase K 200 mg/L, 0.5 % SDS) and incubated for 3 h at 50 °C. DNA was extracted with phenol-chloroform, precipitated in 1/10 volume of NaOAc 2 mol/L and 2 volumes of ethanol at -20 °C overnight, recovered by centrifugation at 1000 × g for 30 min at 4 °C, and then resuspended in TE buffer. RNase A was then added at a concentration of 200 mg/L, then the treated extract was incubated at 37 °C for 30 min and electrophoresed on a 1.2% agarose gel.

### 2.8. Western blot analysis

For Western blotting, cells were washed with ice-cold PBS twice and lysed for 30 min at 4 °C, then debris was removed by centrifugation for 15 min at 15,000×g at 4 °C, and equivalent amounts of protein were separated by 10 % SDS-PAGE and transferred onto nitrocellulose filters. The filters were first stained to confirm uniform transfer of all samples and then incubated in blocking solution for 2 h at room temperature. The filters were reacted firstly with the following antibodies: anti-survivin, anti-bcl and anti-Bax at a dilution of 1:1000 for 2 h, followed by extensive washes with PBS twice and TBST twice. Filters were then incubated with 1:1000 horseradish peroxidase-conjugated secondary antibodies for 1 h, washed with TBST and developed using the Super Signal West Pico Kit. As an internal control, β-actin was detected with anti-β-actin antibodies.

### 2.9. Statistical analysis

All experiments were performed in triplicates and the results were expressed as mean ± SD. Statistical analyses were performed with t-test using SAS 6.12 software.

## 3. Results

### 3.1. Cell viability

**Figure 1.**
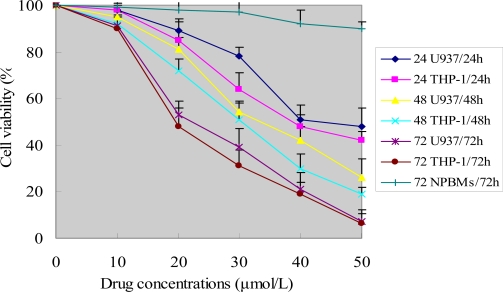
Cell viability caused by ponicidin.

To investigate the growth inhibition effects of ponicidin on leukemia U937 and THP-1 cells as well as normal peripheral blood monocytes (NPBMs), the cells were treated with various concentrations of ponicidin for 0, 24, 48 and 72 h. As shown in Figure 1, ponicidin (over 10 μmol/L) had significant growth inhibition effects on the two kinds of leukemia cells in a dose-and time-dependent manner, while there was no significant cytotoxicity of ponicidin on NPBMs compared to the target leukemia cells. The two kinds of leukemia cells showed somewhat different sensitivity to different concentrations of ponicidin measured by the MTT test, with the THP-1 cells being a bit more sensitive to ponicidin, compared with U937 cells. Cell viability was decreased remarkably after the cells were treated with 50 μmol/L ponicidin for 72 h.

### 3.2. Flow cytometry analysis

**Figure 2.**
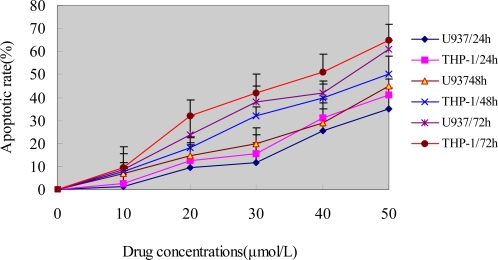
Cell apoptosis caused by ponicidin.

**Figure 3.**
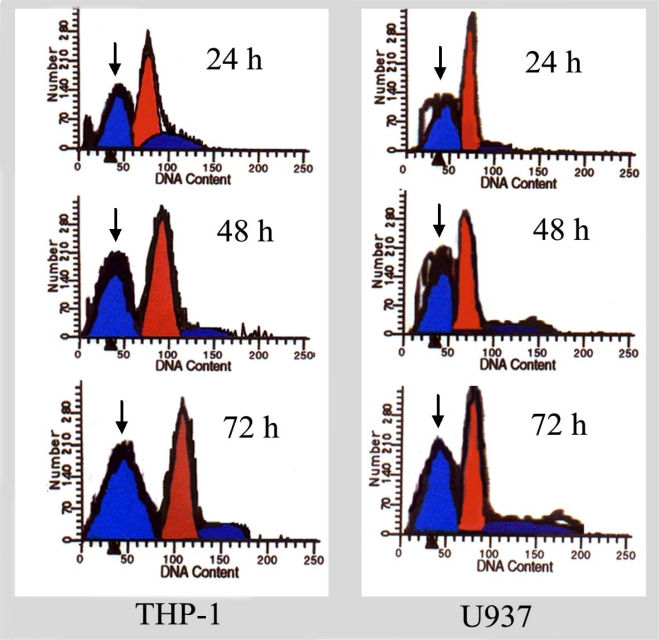
Flow histogram of sub-G1 cells.

To show whether the growth inhibition induced by ponicidin in U937 and THP-1 cells was caused by induction of apoptosis, the cells were stained with PI and analyzed by FCM. As shown in Figure 2, the percentage of sub-G1 cells in U937 and THP-1 cells was increased in a dose- and time dependent manner. Along with the enhancement of cell growth inhibition, apoptotic cells gradually increased and the percentage of sub-G1 cells of both U937 and THP-1 cells reached up to over 60% when the cells were treated with 40 μmol/L ponicidin for 72 h. Flow histogram of sub-G1 cells were also observed clearly after the cells were treated by 50μmol/L ponicidin for different times. As shown in Figure 3, the sub-G1 percentage of both U937 and THP-1 cells gradually increased in a time-dependent manner.

After the cells were treated with different concentrations of ponicidin for 24, 48 and 72 h, the cell viability was determined by MTT assay as described in the Methods. Experiments were done in triplicate.Values represent mean (±SD) cell viability as a percentage of untreated control samples.

After the cells were treated with different concentrations of ponicidin for 24, 48 and 72 h, apoptotic rate was detected as described in the Methods. Cells were stained with PI and then analyzed by FCM, the percentage of sub-G1 cells in the two kinds of cell lines was increased in both time- and dose-dependent manner especially treated with 40 μmol/L ponicidin for 72 h, the percentage of sub-G1 cells of both U937 and THP-1 cells was over 50%. The experiments were repeated three times and the results were presented as mean ±SD.

After the cells were treated by 50 μmol/L ponicidin for different times (24, 48 and 72 h), flow histograms of sub-G1 cells were detected by FCM as described in the Methods. The percentage of sub-G1 cells (shown by the ‘arrow’) was gradually increased in a time-dependent manner in both THP-1 and U937 cells. After treatment of 72 h, the percentage of sub-G1 cells was over 50%.

### 3.3. Expression of apoptosis related genes

**Figure 4.**
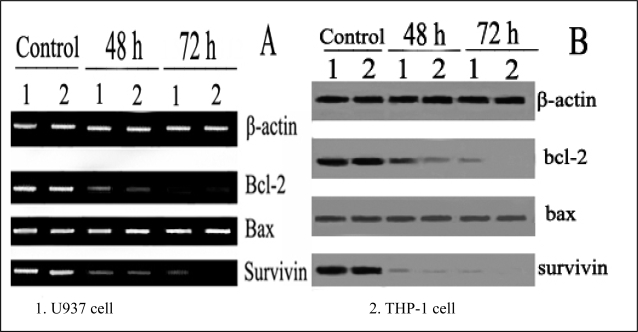
Expression of pro-and anti-apoptotic genes detected by RT-PCR and Western blot. Expression of pro-and anti-apoptotic genes was detected by RT-PCR (A) and Western blot (B) after treatment with 40 μmol/L ponicidin for 48 and 72 h. Both mRNA and protein levels of Survivin and Bcl-2 were down-regulated while the mRNA expression and protein level of Bax remained constant.

After treatment with 40 μmol/L ponicidin for 48 and 72 h, the mRNA expressions of both Survivin and Bcl-2 were down-regulated while mRNA expression of Bax remained constant (Figure 4 A). Likely, the protein levels of Survivin and Bcl-2 were down-regulated and no variation of Bax protein expression was observed concurrently examined by Western blotting when apoptosis occurred (Figure 4B).

### 3.4. Hoechst 33258 staining

**Figure 5.**
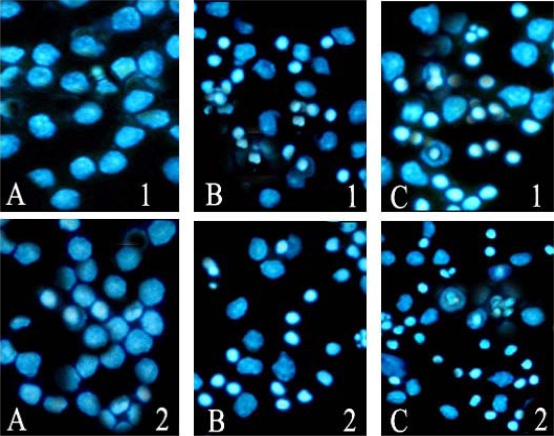
Apoptosis observed by Hoechst 33258 staining (200×). After the cells were treated with 40 μmol/L ponicidin, Hoechst 33258 staining was used to observe the morphological changes of cell apoptosis. Morphological changes of cell apoptosis such as condensation of chromatin and nuclear fragmentations were found clearly. A: Control; B: Cells treated for 48 h; C:Cells treated for 72 h. **1**. U937; **2.** THP-1.

After cells treated with 40 μmol/L ponicidin for 48 and 72 h, marked morphological changes of cell apoptosis such as condensation of chromatin and nuclear fragmentations were found clearly using Hoechst 33258 staining (Fig. 5). Apoptotic cells gradually increased in time-dependent manner in both U937 and THP-1 cells.

### 3.5. DNA fragmentation analysis

**Figure 6.**
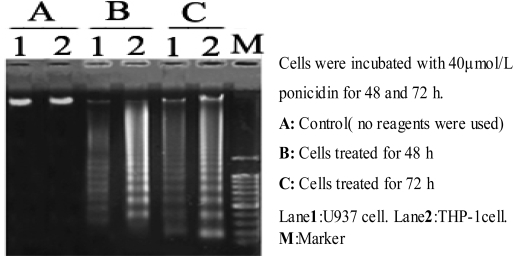
DNA fragmentation analysis.

To observe ponicidin induced apoptosis in monocytic leukemia cells, the integrity of DNA was assessed by agarose gel electrophoresis after the cells were treated for 48 and 72 h. As shown in Fig. 6, incubation of U937 and THP-1 cells with 40 μmol/L ponicidin elicited a characteristic “ladder” of DNA fragments representing integer multiples of the internucleosomal DNA length (about 180∼200 bp). DNA ladder was observed in a dose-manner (Fig. 6).

## 4. Discussion

Despite recent advances in our understanding of the molecular biology of leukemia cells and the induction of some new chemotherapeutic agents for the treatment of this malignant disease, there are few efficient therapeutic measures or regimes, especially for the patients who are in the mid-or final stages, and the dismal 5-year survival rate has not changed too much for this leading cause of cancer deaths in the world [7, 8]. Therefore, it is a permanent subject to find new drugs and effective therapies for the clinical treatment of leukemia. Plant-derived compounds are known to have curative potential.

In this study, we found that ponicidin, a diterpenoid compound extracted from traditional Chinese herbs, could inhibit the proliferation by inducing apoptosis on leukemia U937 and THP-1 cells remarkably when the cells treated with different concentrations of ponicidin (over 10 μmol/L) for different times, and no significant cytotoxicity was found in the normal peripheral blood monocytes. Flow cytometry analysis showed that apoptotic cells gradually increased and the percentage of sub-G1 cells of both U937 and THP-1 cells was over 60% after the cells were treated with 40 μmol/L ponicidin for 72 h, and the suppression was in both time-and dose-dependent manner. Marked morphological changes of cell apoptosis including condensation of chromatin and nuclear fragmentation were observed clearly by Hoechst 33258 staining especially after the cells were treated with ponicidin for 48∼72 h, and typical DNA “ladder” was observed when apoptosis occurred. To clarify the mechanisms of apoptosis caused by ponicidin, we detected the expressions of both mRNA and protein levels of Survivin as well as Bcl-2 and Bax after the cells were treated with 40 μmol/L ponicidin for 48 and 72 h. RT-PCR and Western blot analysis revealed that both Survivin and Bcl-2 expressions were down-regulated remarkably while Bax expression remained constant before and after apoptosis occurred.

Apoptosis play an important role in the development and maintenace of homeostasis with all multicellular organisms, and impaired apoptosis is now recognized to be a key step in tumorigenesis [9]. Recently, inducers of apoptosis have been used in cancer therapy and activation of apoptosis pathways is a key mechanism by which cytotoxic drugs kill tumor cells [10]. All these studies indicate that induction of apoptosis can now be considered an important method of assessment for the clinical effectiveness of many anti-tumor drugs and a significant index for the selection of new anti-tumour drugs [10, 11]. Ponicidin is recently reported to be very effective in inducing growth inhibition and apoptosis in a variety of malignant cell lines. In this study, our results demonstrated that ponicidin has significant anti-proliferation effects by induction of apoptosis on leukemia U937 and THP-1 cells. The data suggest that ponicidin may be used as an effective apoptosis inducer on leukemia cells *in vitro*. These results have not been reported before.

Survivin, a member of apoptosis inhibitor family, is expressed in most human malignancies and implicated in mitosis regulation and preservation of cell viability [12]. Previous data have revealed that survivin is one of the genes most consistently overexpressed in tumor cells which plays important roles in both cell proliferation and cell death [13]. Recent studies [14, 15] have shown that survivin is over expressed and is almost always present in leukemia cells, and expression of survivin always correlates with disease severity and over expression of survivin in tumor cells is a factor of poor prognosis in patients with leukemia. All these data suggest that this protein may play a role in leukemia tumourigenesis. Later studies have demonstrated that survivin is essential for cell cycle progression in leukemia cells, and downregulation of survivin expression may lead to programmed cell death [16], indicating that survivin may be an appealing new target for novel therapies in leukemia [17].

The Bcl-2 family consists of about 20 homologues of important pro- and anti-apoptotic regulators of programmed cell death. Bcl-2 represents the founding member of the new and growing class of cell death inhibiting oncoproteins [18]. The first pro-apoptotic homolog of Bcl-2 family, Bax, was identified by coimmunoprecipitation with Bcl-2 protein. When Bax was overexpressed in cells, apoptotic death in response to death signals was accelerated, earning its designation as a death agonist. When Bcl-2 was overexpressed, it heterodimerized with Bax and death was repressed, thus the ratio of Bcl-2 to Bax is important in determining susceptibility to apoptosis [19, 20]. In this study, our results revealed that Bcl-2 expression was down-regulated remarkably in ponicidin induced apoptosis on leukemia cells. Though Bax expression remained constant before and after apoptosis ocurred, the ratio of Bcl-2 to Bax was downregulated, apoptosis therefore was induced in ponicidin treated leukemia cells.

Though survivin and Bcl-2 are both apoptosis inhibitors, they function in the regulation of cell apoptosis through different pathways. Bcl-2 regulates apoptosis mainly by preventing cytochrome C release from mitochondrion to cytoplasm [18, 19], whereas survivin acts by direct inhibition of the terminal effector proteases of apoptosis. Studies have demonstrated that survivin could directly inhibit the activities of Caspase-3 and 7, and block the process of apoptosis [21]. Previous studies demonstrated that the expression of survivin was significantly associated with Bcl-2 expression and the expression of survivin, in conjunction with Bcl-2, might cause more pronounced anti-apoptotic effects [22, 23]. Therefore the expression of survivin in cooperation with Bcl-2 is a significant prognostic parameter and a new therapeutic target in cancer [24, 25]. These data indicated that the coexpression of survivin and Bcl-2 may play an important role in the regulation of cancer cell apoptosis. In the present study, we found that the expressions of both survivin and Bcl-2 were downregulated concurrently in ponicidin induced apoptosis on the two leukemia cell lines. Our results agree with the findings of previous investigations, which showed that the coexpressions of survivin and Bcl-2 play an important role in drug induced apoptosis [22, 23]. The data suggest that ponicidin may serve as a potential therapeutic agent for leukemia. The *in vivo* antitumor effects of ponicidin as well as its potential clinical effectiveness need further and profound investigation.

## 5. Conclusions

Ponicidin has significant anti-proliferation effects by inducing apoptosis on leukemia cells *in vitro*, downregulation of survivin as well as Bcl-2 expressions may be the important apoptotic inducing mechanisms. The results suggest that ponicidin may serve as potential therapeutic agents for leukemia. To our knowledge, this is the first report about the roles of ponicidin on monocytic leukemia cell *in vitro*.

## Figures and Tables

**Table 1.** The PCR primers used in this study.

β-actin **mRNA** (243 bp)	
Sense primer	5′-CTTCTACAATGAGCTGCGTG-3′
Anti-sense primer:	5′-TCATGAGGTAGTCAGTCAGG -3′
Survivin **mRNA** (393 bp)	
Sense primer :,	5′-CTTTCTCAAGGACCACCGCATC-3′
Anti-sense primer :	5′-CAATCCATGGCAGCCAGCTGC-3′
bcl-2 **mRNA** (478 bp) :	
Sense primer:	5′-CTACGAGTGGGATGCGGGAGATG-3′
Sense primer :	5′-GGTTCAGGTACTCAGTCATCCACAG-3′
Bax **mRNA** (258 bp) :	
Sense primer:	5′-ACCAA GAA GCTGA GCGA GTGTC-3′
Anti-sense primer:	5′-TGTCCA GCCCA TGA TGGTTC-3′
